# Clinical outcomes of 1PN- and 3PN-derived blastocysts in the absence of transferable 2PN embryos: A retrospective analysis

**DOI:** 10.1371/journal.pone.0355689

**Published:** 2026-08-24

**Authors:** Nuri Peker, Ayşen Yücetürk, Esra Tığlı, Özge Karaosmaoğlu, Burak Elmas, İlke Özer Aslan, Bülent Tıraş

**Affiliations:** 1 Department of Obstetrics and Gynecology, Reproductive Endocrinology, Acıbadem Maslak Hospital, İstanbul, Türkiye; 2 Department of Obstetrics and Gynecology, Kütahya Health Sciences University, Kütahya, Türkiye; 3 Department of Obstetrics and Gynecology, Reproductive Endocrinology, Acıbadem Mehmet Ali Aydınlar University, Faculty of Medicine, İstanbul, Türkiye; University of Connecticut, UNITED STATES OF AMERICA

## Abstract

**Objective:**

We aimed to determine the blastocyst formation rates, preimplantation genetic testing for aneuploidy (PGT-A) results, and pregnancy rates of embryos derived from 1pronucleus (1PN) and 3 pronuclei (3PN).

**Methods:**

A total of 150 patients who developed 1PN and/or 3PN embryos were enrolled in the study. The embryos were divided into three groups according to the number of pronuclei: group (i) consisted of 1PN embryos; group (ii) consisted of 3PN embryos; and group (iii) was the control group, which included 2PN embryos. The primary outcomes were the blastocyst formation rate and euploidy rate of the 1PN and 3PN embryos. The secondary outcomes were pregnancy and live birth rates after embryo transfer.

**Results:**

A total of 1711 (72.1%) metaphase II (MII) oocytes were obtained among 2373 oocytes. A total of 508 day-5 embryos developed, of which 44 (8.7%) were identified as 3PN, 13 (2.6%) as 1PN, and 451 (88.8%) as 2PN. PGT-A was performed on 23 3PN embryos and 4 1PN embryos. Overall comparison revealed a significant difference among groups (chi-square (χ²) = 51.11, df = 2, p < 0.0001). In pairwise analyses, 1PN zygotes had significantly lower blastocyst formation rates than both 2PN and 3PN zygotes (both p < 0.0001), whereas the difference between 2PN and 3PN zygotes was not significant (p = 0.5025). All tested blastocysts were reported as aneuploid and none of these embryos were transferred. A total of five 3PN blastocyst transfers were performed in five patients, one 1PN blastocyst transfer was performed in one patient, and combined 1PN and 3PN blastocyst transfer was performed in one patient. Importantly, no preimplantation genetic testing for aneuploidy (PGT-A) was performed on the embryos selected for transfer, and all transfers were carried out without genetic testing. The only pregnancy was achieved in the patient who underwent combined transfer of 1PN and 3PN blastocysts.

**Conclusion:**

Blastocyst formation and pregnancy rates derived from abnormally fertilized embryos are low; however, transfer of blastocysts derived from 1PN and 3PN embryos may be considered in patients who do not develop transferable 2PN blastocysts.

## Introduction

Successful fertilization requires the fusion of male and female gametes. In in vitro fertilization (IVF) and intracytoplasmic sperm injection (ICSI) cycles, this event is morphologically confirmed by the presence of two pronuclei (2PN) 16–20 hours after insemination [1]. However, pronuclei abnormalities may occur including 0PN, 1PN, 3PN or multiple PN, which may indicate failed or abnormal fertilization. A 0PN embryo is the absence of pronuclei 16–20 hours after insemination, indicating that PN occurs before or after this time period [2,3]. Despite this, good-quality blastocyst development may occur from 0PN zygotes, and healthy live births have been reported [3]. In contrast, 1PN and 3PN embryos, as well as embryos with multiple pronuclei, are considered pathological and rarely develop into good-quality embryos [2,4,5]. Abnormal pronuclear formation, particularly the presence of ≥3 pronuclei, has been strongly associated with increased multinucleation at the two-cell stage, suggesting that defects arising at fertilization may propagate into early mitotic divisions and compromise nuclear integrity in developing embryos [4]. Lebovitz et al. compared the development performance of 1PN embryos with that of 2PN embryos and reported that 1PN embryos have low development potential. Furthermore, they investigated the chromosomal structure of 1PN embryos and reported significantly lower euploidy rates compared with 2PN embryos [5]. The blastocyst formation rate and top-quality blastocyst rate were 24% and 22.3%, respectively, whereas corresponding rates of 37.9% and 48.1% were reported for 2PN embryos [5].

Conversely, some studies have reported that blastocysts derived from 1PN or 3PN embryos may be chromosomally normal and could be considered for embryo transfer when no transferable 2PN-derived blastocyst is available [6,7]. Mutia et al. reported a study investigating the aneuploidy rates of 3PN embryos. Thirty 3PN embryos were analyzed, and abnormal chromosomal findings were observed in 66.7% of embryos, including triploidy (43.3%), mosaicism (13.4%), and aneuploidy (10%); the remaining 33.3% were chromosomally normal, supporting the view that selected 3PN embryos may retain developmental competence [6]. In another study, Wang et al. compared 0PN- and 1PN-derived blastocysts with 2PN-derived blastocysts and reported similar aneuploidy rates among the groups. Following genome-wide ploidy and haplotyping analysis, they reported three live births from five transfers (60%) of 0PN- and 1PN-derived euploid blastocysts, and suggested that 0PN- and 1PN-derived embryos can be considered for transfer in patients who do not develop transferable 2PN blastocysts [7].

Although the developmental competence of 1PN- and 3PN-derived embryos has been investigated in several studies, clinical outcomes in patients whose only transferable blastocysts originate from abnormally fertilized zygotes remain poorly characterized. In this retrospective cohort of 150 ICSI patients, we therefore aimed to (i) determine blastocyst formation rates across 1PN, 2PN, and 3PN zygotes; (ii) report PGT-A outcomes of the resulting 1PN- and 3PN-derived blastocysts; and (iii) describe pregnancy and live birth outcomes following transfer of these embryos when no transferable 2PN-derived blastocyst was available.

## Materials & methods

This study was conducted between October 15, 2025, and November 15, 2025, at the Acıbadem Maslak Hospital IVF Center, and during this one‑month period, the medical records of patients who had undergone treatment at our clinic between January 1, 2022, and January 1, 2025, were retrospectively reviewed. Clinical records of 8,000 patients who underwent IVF treatment, 150 patients who developed 1PN and/or 3PN embryos after ICSI were included in the study. The study was conducted in accordance with the Declaration of Helsinki, and the study protocol was approved by the local institutional review board and ethics committee (Approval No.2025-14/550). Written informed consent was obtained from all patients.

The embryos were divided into three groups: group (i), consisting of 1PN embryos; group (ii), consisting of 3PN embryos; and group (iii), which served as the control group and included 2PN embryos. The primary outcomes of the study were the blastocyst formation rates and euploidy rates of the 1PN and 3PN embryos. The secondary outcome of the study was the pregnancy rate after embryo transfer.

### ICSI procedure and embryo culture

All 150 included cycles were fertilized by intracytoplasmic sperm injection (ICSI); conventional IVF cycles were excluded. Fertilization was assessed as a single static observation 16–20 hours after ICSI under an inverted microscope. Embryos were cultured in Irvine Single Step NXC medium (Irvine Scientific, Santa Ana, CA, USA) under mineral oil in benchtop incubators at 37 °C, 6.0–6.5% CO2 and 5% O2, maintaining a culture pH of 7.29–7.32, until day 5. Of the 1,711 metaphase II (MII) oocytes, a total of 1,487 zygotes were included in the final analysis. The difference between these values is attributable to the exclusion of degenerated oocytes and zygotes with abnormal pronuclear patterns, including 0PN and multiple PN. Specifically, 31 degenerated oocytes and 193 zygotes with abnormal pronuclear status (186 0PN and 7 multiple PN) were excluded from the analysis. Blastocyst morphology on day 5 was assessed according to the Gardner and Schoolcraft grading system, based on expansion degree (1–6), inner cell mass quality (A-C), and trophectoderm quality (A-C). When PGT-A was performed, trophectoderm biopsy was carried out on day-5 blastocysts, and the biopsied cells were analyzed by next-generation sequencing (NGS).

In our study, embryos derived from 2PN zygotes were prioritized in PGT-A and embryo transfer. In patients with a sufficient number of 2PN-derived blastocysts, PGT-A and embryo transfer were not performed on 1PN- or 3PN-derived blastocysts. In contrast, in patients without 2PN-derived blastocysts or with a low number of 2PN-derived blastocysts, 1PN- and/or 3PN-derived embryos were subjected to PGT-A. None of the embryos diagnosed as aneuploid by PGT‑A were transferred among the patients. Furthermore, transfer of 1PN- and/or 3PN-derived blastocysts was considered only in patients in whom no 2PN-derived blastocysts were available or in cases where pregnancy was not achieved after transfer of 2PN-derived blastocysts and no remaining 2PN embryos were available.

### Statistical analysis

Analyses were performed using SPSS Statistics version 30.0 (IBM Corp., Armonk, NY) and GraphPad Prism version 9.0 (GraphPad Software, San Diego, CA). Continuous variables were assessed for normality with the Shapiro-Wilk test. Age and BMI are reported as mean ± SD in line with standard reporting practice, while skewed variables (E2, total oocyte number, MII number, and per-PN zygote counts) are reported as median with interquartile range (IQR). Blastocyst formation rates across PN groups were compared at the zygote level using the chi-square (χ²) test of homogeneity, with pairwise comparisons by two-sided Fisher's exact test and Bonferroni correction for three comparisons (adjusted α = 0.0167). Ninety-five per cent confidence intervals (95% CI) were calculated using the Wilson score method. Other categorical variables (preimplantation genetic testing for aneuploidy (PGT-A) outcomes, pregnancy, and live birth) were compared using Fisher's exact test. A post-hoc power analysis (G*Power 3.1) confirmed that the sample of 1,487 zygotes provided >99% power to detect the observed effect (Cohen's w = 0.185) at α = 0.05. All tests were two-sided, and p < 0.05 was considered significant.

## Results

A total of 150 patients were enrolled in the study. Baseline and cycle characteristics of the study cohort are summarized in Table 1. The mean age of the patients was 35.8 ± 6.2 years, and the mean BMI of the cohort was 26.1 ± 5.3 kg/m². The median estradiol (E_2_) level was 2098 pg/mL (interquartile range [IQR], 1293–3453), the median total oocyte number was 14 (IQR, 8–20), and the median metaphase II (MII) oocyte number was 9 (IQR, 6–15). Overall, 1,711 (72.1%) MII oocytes were obtained from 2,373 retrieved oocytes.

**Table 1:** Baseline demographic and cycle characteristics of the study cohort (N = 150).

Variable	Value
Age (Years)	35.8 ± 6.2
BMI (kg/m²)	26.1 ± 5.3
E_2_ Level (pg/mL)	2098 (1293–3453)
Total Oocyte Number	14 (8–20)
MII number	9 (6–15)

Data are presented as mean ± SD (age, BMI) or median (interquartile range) (E2 level, oocyte counts), as appropriate. BMI, body mass index; E2, estradiol; MII, metaphase II.

**Fig 1:**
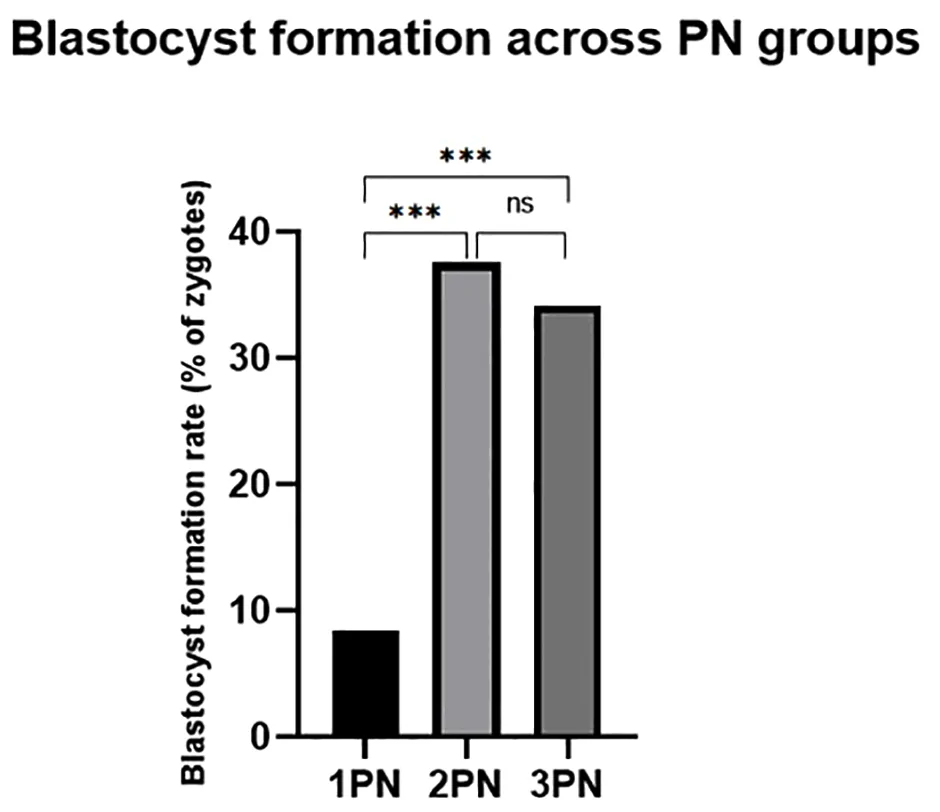
Blastocyst formation rates according to pronuclear status. Blastocyst formation occurred in 13/154 (8.4%) 1PN zygotes, 451/1204 (37.5%) 2PN zygotes, and 44/129 (34.1%) 3PN zygotes. Overall comparison among groups was significant (χ² = 51.11, df = 2, p < 0.0001). In pairwise comparisons, 1PN zygotes had significantly lower blastocyst formation rates than both 2PN and 3PN zygotes (both p < 0.0001), whereas the difference between 2PN and 3PN zygotes was not significant (p = 0.5025). ***p < 0.001; ns, not significant.

Blastocyst formation rates differed significantly according to pronuclear status. Formation rates were 8.4% (13/154) for 1PN zygotes, 37.5% (451/1204) for 2PN zygotes, and 34.1% (44/129) for 3PN zygotes (Fig 1). Overall comparison revealed a significant difference among groups (chi-square (χ²) = 51.11, df = 2, p < 0.0001). In pairwise analyses, 1PN zygotes had significantly lower blastocyst formation rates than both 2PN and 3PN zygotes (both p < 0.0001), whereas the difference between 2PN and 3PN zygotes was not significant (p = 0.5025).

Of the 508 blastocysts obtained, 451 (88.8%) originated from 2PN zygotes, 44 (8.7%) from 3PN zygotes, and 13 (2.6%) from 1PN zygotes. Tables 2 and 3 present the numbers of blastocysts developed from 3PN and 1PN embryos, respectively, together with their corresponding PGT-A results. PGT-A was performed on 23 3PN-derived blastocysts and 4 1PN-derived blastocysts, and all tested embryos were reported as aneuploid. Detailed chromosomal findings of the tested embryos are summarized as follows: among the 23 PGT-A-tested 3PN-derived blastocysts, the most frequent finding was complex aneuploidy involving three or more chromosomes (n = 13; 56.5%), followed by single whole-chromosome aneuploidy (n = 4; 17.4%), multiple whole-chromosome aneuploidies (n = 3; 13.0%), and segmental or partial aneuploidy (n = 3; 13.0%; including one mosaic monosomy). Among the 4 PGT-A–tested 1PN-derived blastocysts, findings included complex aneuploidy, multiple whole-chromosome aneuploidies, single monosomy, and segmental aneuploidy in one embryo each. No euploid embryo was identified in either group (0/27; 0%). Notably, none of the 3PN-derived blastocysts exhibited a triploid (69, XXX or 69, XXY) chromosomal profile. Embryos identified as aneuploid by PGT-A were not transferred.

**Table 2:** Characteristics, blastocyst development, and preimplantation genetic testing for aneuploidy (PGT-A) results of embryos derived from 3PN zygotes.

Age	BMI	E_2_ Level	Total Oocyte Number	MII number	3pn	Blastocyst number/ Grade	PGT-A Results
40–44	25.7	–	6	4	1	1 (4AB)	Aneuploid
35–39	35	3560	20	16	2	2 (4AA. 3BB)	Aneuploid
30–34	34.1	1450	7	5	1	1(3AA)	Aneuploid
30–34	30.2	1425	16	9	1	1 (2BC)	Aneuploid
30–34	23.4	–	17	12	1	1 (3BC)	Aneuploid
40–44	23.5	1760	9	8	1	1 (5AA)	Aneuploid
35–39	22.8	–	26	20	1	1 (5BB)	Aneuploid
≥45	37.4	1297	6	5	1	1 (5BB)	Aneuploid
35–39	29.3	4515	23	13	1	1 (4BB)	Aneuploid
<30	24.6	–	36	33	3	1 (3BC)	Aneuploid
35–39	31.2	7236	39	15	1	1 (3BC)	Aneuploid
30–34	24.8	_	13	8	1	1 (3BC)	Aneuploid
40–44	19.8	2230	9	7	1	1 (EB)	Aneuploid
40–44	22.6	1468	10	9	2	2 (3BC, 3BC)	Aneuploid
40–44	25.8	1678	11	9	1	1 (3AB)	Aneuploid
35–39	24.6	3450	17	13	2	1 (3AA)	Aneuploid
≥45	31.2	428	3	2	1	1 (3BC)	Aneuploid
40–44	36.5	–	6	4	1	1 (3BC)	Aneuploid
<30	22.3	2511	23	10	1	1 (2BB)	Aneuploid
40–44	28.3	1450	10	9	1	1 (3AB)	Aneuploid
35–39	27	340	2	2	1	1 (2BC)	Aneuploid

**Table 3:** Characteristics, blastocyst development, and preimplantation genetic testing for aneuploidy (PGT-A) results of embryos derived from 1PN zygotes.

Age	BMI (kg/m^2)^	E_2_ Level (pg/mL)	Total Oocyte Number	MII number	1PN	Blastocyst number/ Grade	PGT-A Results
30–34	27.6	1980	15	8	1	1(2BA)	Aneuploid
40–44	19.9	2319	15	13	2	1 (3BC)	Aneuploid
<30	36.4	_	22	19	5	1 (5AA)	Aneuploid
<30	22.3	_	6	4	1	1 (2BC)	Aneuploid

Table 4 shows the number and morphological grades of transferred 1PN- and/or 3PN-derived blastocysts, together with the corresponding pregnancy outcomes. A 3PN-derived blastocyst transfer was performed in five patients, a 1PN-derived blastocyst transfer was performed in one patient, and a combined transfer of a 1PN-derived blastocyst (4BC) and a 3PN-derived blastocyst (4AA) was performed in one patient. Importantly, no PGT-A testing was performed on the embryos selected for transfer; therefore, their chromosomal status remained unknown. The only pregnancy was achieved after combined transfer of 1PN- and 3PN-derived blastocysts. The patient was followed throughout pregnancy and delivered a healthy male infant weighing 3,700 g at 37 weeks of gestation

**Table 4:** Transferred 1PN- and/or 3PN-derived blastocysts, embryo grades, and pregnancy outcomes.

Age	Total Oocyte number	MII number	2PN number	2PN-Blastocyst number/grade	3PN number	1PN number	Blastocyst (3PN-1PN) number/ grade	Pregnancy
≥45	21	19	10	0	4	0	1 (5AB) (3PN)	no
≥45	6	5	3	0	1	0	1 (4BC) (3PN)	no
≥45	2	2	1	0	1	0	1 (3BB) (3PN)	no
<30	15	12	7	0	0	1	1 (EB) (1PN)	no
<30	9	6	4	0	1	1	4AA (3PN) 4BC (1PN)	yes
30–34	18	14	7	3 (5AA. 5AA. 4BC)	1	0	1 (5AA) (3PN)	no
≥45	9	5	1	1 (4BC)	1	0	1 (3AA) (3PN)	no

*2PN embryos were transferred before the 3PN embryo transfer, but pregnancy was not achieved.

## Discussion

Embryos arising from abnormal fertilization including 0PN, 1PN, and 3PN zygotes are generally considered discard embryos and are not transferred in routine clinical practice. However, their developmental and chromosomal potential has attracted growing attention, particularly in patients who do not develop transferable 2PN-derived blastocysts. In the present retrospective cohort of 150 ICSI patients, we therefore evaluated the blastocyst formation rates, PGT-A outcomes, and clinical results of embryos derived from 1PN and 3PN zygotes. Although 3PN-derived zygotes reached the blastocyst stage at a rate comparable to that of 2PN, all 27 PGT-A–tested 1PN- and 3PN-derived blastocysts were aneuploid, and only a single live birth was achieved among seven transfers of these embryos.

Zygotes lacking visible pronuclei 16–20 hours after ICSI or C-IVF are defined as 0PN and classified as abnormally fertilized oocytes; however, healthy live births following 0PN embryo transfer have been reported in several studies [3,8,9]. Liu et al. published a retrospective study including 4424 IV cycles and reported 13 live births after the transfer of 275 0PN embryos [3]. In another related study, Maria Valeria Paz et al. reported the genetic and clinical outcomes of 0PN embryos. In this study, they reported 48% implantation rate and a 50% ongoing pregnancy rate following the transfer of 27 0PN embryos, ultimately resulting in 13 live births. Moreover, blastocyst biopsy was performed on 17 0PN embryos, resulting in seven euploid and ten aneuploid embryos [8]. Similarly, Fu Lei et al. investigated the developmental potential of 0PN embryos and reported similar implantation, pregnancy and live birth rates comparable to those of normally fertilized embryos [9].

The transfer of 1PN and 3PN blastocysts has been discussed in patients with diminished ovarian reserves and is recommended only in patients with a lack of high-quality 2PN blastocyst development. Nevertheless, several studies have reported that 1PN and 3PN embryos can result in successful pregnancies and live births [10–13]. Wang et al. assessed live birth rates following the transfer of embryos derived from 1PN zygotes. A total of 266 vitrified–warmed 1PN embryos were classified into three groups according to the Gardner morphological grading system, and good-quality blastocysts (3–6AA, 3–6BA, and 3–6AB) were reported to result in live births, with a rate of 48.7%. Furthermore, Wang et al. highlighted the critical importance of the inner cell mass and trophectoderm cells for predicting live birth rates in 1PN embryos [10]. Si J. investigated the obstetrical and neonatal outcomes of 1PN embryos and reported similar live birth rates between day-5 1PN and 2PN embryos. As the result, no significant differences were observed in miscarriage rates, congenital malformations, or psychomotor development [11]. Similarly, Hondo et al. conducted a study and reported that 1PN embryos can be transferred, particularly in patients with ICSI [12]. They reported that the live birth rates were similar to those of 2PN embryos obtained via conventional IVF; however, the blastocyst rates were significantly lower in 1PN embryos obtained via ICSI [12]. The chromosomal constitution of 1PN and 3PN embryos has been evaluated in several studies, with some embryos demonstrating normal karyotypes [13–16]. Bradley et al. performed PGT-A on 1PN embryos, which were classified as (i) embryos obtained via ICSI and (ii) embryos obtained via conventional IVF. Blastocyst development rates of 1PN embryos were compared with those of 2PN embryos and were reported as 14.8% versus 36.4% after IVF and 6.6% versus 34.0% after ICSI. Furthermore, PGT-A was performed, and the chromosomal aneuploidy rates were similar, at 39.7% and 40.6% [13]. Following the transfer of PGT-normal embryos, nine pregnancies were achieved. Hirata et al. conducted a study demonstrating the CGH array results of the 1pn-3pn embryos and reported an aneuploidy rate of 30.8% in 1PN IVF and 33.3% in 1pn ICSI [14]. On the other hand, clinical outcomes following the transfer of 1PN- and 3PN-derived embryos across studies are markedly heterogeneous. Reichman et al. evaluated zygotes that were initially identified as 2PN but transitioned to 1PN or 3PN before cleavage, and reported implantation and viable pregnancy rates of 6.4% and 1.3%, respectively, for these 1PN zygotes, with no implantations among nine transferred 3PN zygotes [15]. Capalbo et al. applied a ploidy-aware PGT-A approach to abnormally fertilized oocytes and demonstrated that most 1PN- and 2.1PN-derived blastocysts were in fact diploid (69.2% and 85.7%, respectively); three live births were achieved from such embryos (one 1PN and two 2.1PN origin), supporting the feasibility of rescuing selected abnormally fertilized blastocysts [16]. Between 1999 and 2021, various articles demonstrating live births after the transfer of 1PN embryos were published. The live birth rates reported in these studies were between 13% and 32.1% [13–18]. The limitations of all the studies mentioned above were that the embryos were examined under a microscope with one static observation between 16–20 hours after ICSI, and the embryos could not follow with a time-lapse embryoscope. In our study, we performed PGT-A on only 4 of the 13 1PN embryos, and all of them were aneuploid.

A 3PN embryo results from abnormal fertilization and is defined by the presence of three pronuclei 16–20 hours after ICSI or conventional IVF. Polyspermic fertilization, particularly in conventional IVF, or meiotic failure of the oocyte are considered the primary underlying mechanisms [19]. Such pregnancies often result in spontaneous abortion during early gestation [19]. The incidence of 3PN zygotes ranges from 5.0% to 8.1% in conventional IVF cycles and from 2.5% to 6.2% in ICSI cycles [5]. In a study by Ziljiang Chen et al., the diploid status of 3PN embryos was assessed, demonstrating a higher proportion of diploid embryos in ICSI-derived 3PN embryos compared with those derived from conventional IVF (25% vs. 3.85%, respectively). Furthermore, they demonstrated that the additional pronucleus in 3PN embryos originated from the second polar body and therefore may not contain genetic material [20].

Another proposed mechanism involves micro-pronuclei fragmented from a normally fertilized 2PN zygote, which may be misidentified as 3PN under microscopic evaluation and are referred to as micro-3PN or 2.1PN embryos [20]. Embryos classified as 2.1PN have been reported to retain developmental potential, including the ability to form normal blastocysts and euploid embryos, ultimately resulting in live births [21]. Yalçınkaya et al. reported a case report of 3PN embryo transfer resulting in live birth after confirmation of euploidy with PGT-A [22]. However, data regarding live birth rates following 3PN embryos remain limited. In contrast to the studies mentioned above, Takahashi et al. conducted a retrospective cohort study comparing 2.1PN embryos with 2PN embryos and reported that although 2.1PN embryos can reach the blastocyst stage and may be diploid, they exhibit high aneuploidy rates and should not be transferred [23]. Although the rates of blastocyst formation were comparable to those of 2PN embryos (43.8% vs. 54.8%), 13 out of 15 2.1PN blastocysts that were aneuploid could not be transferred [23]. Our findings were similar to those reported by Takahashi et al. In our study, we performed PGT-A on 23 3PN embryos, all of which were aneuploid. Furthermore, we transferred exclusively five 3PN-derived blastocysts in five patients without prior PGT-A, none of which resulted in pregnancy. Consequently, no pregnancies were achieved following transfer of 3PN embryos in our study. One noteworthy observation in our cohort is that none of the 23 PGT-A–tested 3PN-derived blastocysts exhibited a triploid chromosomal profile, despite triploidy being reported as the most frequent abnormality (43.3%) among 3PN-derived embryos by Mutia et al. [6]. Several findings in the published literature are relevant to this observation. Capalbo et al. demonstrated that standard copy-number–based PGT-A cannot reliably distinguish ploidy states, and that dedicated SNP allele-ratio analysis on the same trophectoderm biopsy (1:1 = diploid, 2:1 = triploid) is required for this purpose; when such ploidy assessment was applied, most 1PN- and 2.1PN-derived blastocysts were in fact diploid (69.2% and 85.7%, respectively), and three live births were achieved from these embryos [16]. Chen et al. further showed that ICSI-derived 3PN zygotes more frequently display a diploid chromosomal constitution than their conventional-IVF counterparts (25% vs 3.85%), likely because the additional pronucleus may originate from the second polar body and carry no genetic material [20]. These considerations suggest that the absence of triploidy in our 3PN-derived blastocysts may reflect both the biological features of ICSI-derived 3PN zygotes and the detection limits of copy-number–based NGS PGT-A. Taken together, the absence of detected triploidy in our cohort may reflect both methodological limitations of copy-number–based PGT-A and biological heterogeneity of 3PN embryos, rather than a true absence of triploidy.

### Limitations of the study

This study has several limitations. First, its retrospective design and the relatively small number of 1PN- and 3PN-derived blastocysts limit the generalizability of the findings. Second, pronuclear assessment was based on a single static observation performed 16–20 hours after ICSI, and therefore dynamic developmental events that could have been captured by time-lapse monitoring were not available. Third, not all 1PN- and 3PN-derived blastocysts underwent PGT-A, because in patients with an adequate number of 2PN-derived blastocysts, priority was given to embryos of normal fertilization status for genetic testing and transfer. As a result, the true euploidy potential of all abnormal-pronuclear blastocysts in this cohort could not be fully assessed. Fourth, in the only case resulting in live birth, two blastocysts were transferred simultaneously. Therefore, the embryo of origin of the successful pregnancy cannot be determined with certainty. Because no postnatal genetic confirmation was performed, any conclusion regarding whether the live birth originated from the 1PN- or 3PN-derived blastocyst remains speculative. In addition, the patient in this case was 25 years old, which may have favorably influenced implantation and live birth potential. Therefore, the observed outcome cannot be interpreted independently of maternal age, embryo morphology, and the limited sample size. Future prospective studies incorporating time-lapse embryoscopy and embryo-of-origin confirmation are needed.

## Conclusion

Several studies have reported the development of good-quality euploid embryos from abnormally fertilized zygotes, resulting in pregnancies and live births, in some cases with outcomes comparable to those of 2PN blastocyst transfer. However, in contrast to these reports, our study yielded only one pregnancy and live birth following combined transfer of 1PN- and 3PN-derived embryos among the 57 blastocysts obtained from zygotes with pronuclear abnormalities. Thus, embryos exhibiting pronuclear abnormalities, including 1PN and 3PN embryos, appear to have limited potential for euploidy, pregnancy, and live birth; however, transfer of 1PN- and/or 3PN-derived embryos may be considered in selected patients who do not have any transferable 2PN-derived blastocysts.

## Supporting information

S1 Data (XLSX)

## Abbreviations

PGT-A: preimplantation genetic testing for aneuploidy
PN: pronuclei
IVF: in vitro fertilization
MII: metaphase 2
ICSI: intracytoplasmic sperm injection
C-IVF: conventional in vitro fertilization
DOR: diminished ovarian reserve
DHEA: dihydroepiandrosterone
PRP: platelet rich plasma
IVM: in vitro maturation.

## References

[1] Otsu E, Sato A, Nagaki M, Araki Y, Utsunomiya T. Developmental potential and chromosomal constitution of embryos derived from larger single pronuclei of human zygotes used in in vitro fertilization. Fertil Steril. 2004;81(3):723–4. doi: 10.1016/j.fertnstert.2003.07.042 15037434

[2] Kobayashi T, Ishikawa H, Ishii K, Sato A, Nakamura N, Saito Y, et al. Time-lapse monitoring of fertilized human oocytes focused on the incidence of 0PN embryos in conventional in vitro fertilization cycles. Sci Rep. 2021;11(1):18862. doi: 10.1038/s41598-021-98312-1 34552114 PMC8458381

[3] Liu J, Wang XL, Zhang X, Shen CY, Zhang Z. Live births resulting from 0PN-derived embryos in conventional IVF cycles. J Assist Reprod Genet. 2016;33(3):373–8. doi: 10.1007/s10815-015-0644-6 26749389 PMC4785154

[4] Li M, Xue X, Shi J. High proportion of zygotes with multiple pronuclei increase the embryo multinucleation rate during conventional IVF. Sci Rep. 2024;14(1):26497. doi: 10.1038/s41598-024-78342-1 39489843 PMC11532555

[5] Lebovitz O, Noach-Hirsh M, Taieb S, Haas J, Zilberberg E, Nahum R, et al. Embryos derived from single pronucleus are suitable for preimplantation genetic testing. Fertil Steril. 2024;122(4):598–606. doi: 10.1016/j.fertnstert.2024.05.152 38788891

[6] Mutia K, Wiweko B, Iffanolida PA, Febri RR, Muna N, Riayati O. The Frequency of Chromosomal Euploidy Among 3PN Embryos. J Reprod Infertil. 2019;20(3):127–31. 31423415 PMC6670263

[7] Wang J, Xie H, Zou Y, Gao M, Wang L, Liu X, et al. The use of blastocysts developing from nonpronuclear and monopronuclear zygotes can be considered in PGT-SR: a retrospective cohort study. BMC Pregnancy Childbirth. 2025;25(1):530. doi: 10.1186/s12884-025-07621-0 40319238 PMC12049007

[8] Paz MV, Chiera M, Hovanyecz P, Cicaré J, Perfumo P, Domenech L, et al. Blastocysts Derived From 0PN Oocytes: Genetic And Clinical Results. JBRA Assist Reprod. 2020;24(2):143–6. doi: 10.5935/1518-0557.20190084 32202747 PMC7169913

[9] Fu L, Zhou W, Li Y. Development and frozen-thawed transfer of non-pronuclear zygotes-derived embryos in IVF cycles. Eur J Obstet Gynecol Reprod Biol. 2021;264:206–11. doi: 10.1016/j.ejogrb.2021.07.033 34329946

[10] Wang T, Si J, Wang B, Yin M, Yu W, Jin W, et al. Prediction of live birth in vitrified-warmed 1PN-derived blastocyst transfer: Overall quality grade, ICM, TE, and expansion degree. Front Physiol. 2022;13:964360. doi: 10.3389/fphys.2022.964360 36439241 PMC9685792

[11] Si J, Zhu X, Lyu Q, Kuang Y. Obstetrical and neonatal outcomes after transfer of cleavage-stage and blastocyst-stage embryos derived from monopronuclear zygotes: a retrospective cohort study. Fertil Steril. 2019;112(3):527–33. doi: 10.1016/j.fertnstert.2019.04.045 31272721

[12] Hondo S, Arichi A, Muramatsu H, Omura N, Ito K, Komine H, et al. Clinical outcomes of transfer of frozen and thawed single blastocysts derived from nonpronuclear and monopronuclear zygotes. Reprod Med Biol. 2019;18(3):278–83. doi: 10.1002/rmb2.12275 31312107 PMC6613012

[13] Bradley CK, Traversa MV, Hobson N, Gee AJ, McArthur SJ. Clinical use of monopronucleated zygotes following blastocyst culture and preimplantation genetic screening, including verification of biparental chromosome inheritance. Reprod Biomed Online. 2017;34(6):567–74. doi: 10.1016/j.rbmo.2017.03.013 28431828

[14] Hirata K, Goto S, Izumi Y, Taguchi M, Hayashi A, Fujioka M, et al. Chromosome analysis of blastocysts derived from single pronuclear zygotes by array CGH and clinical outcomes by the transfer of single pronuclear zygotes. J Assist Reprod Genet. 2020;37(7):1645–52. doi: 10.1007/s10815-020-01800-y 32415641 PMC7376778

[15] Reichman DE, Jackson KV, Racowsky C. Incidence and development of zygotes exhibiting abnormal pronuclear disposition after identification of two pronuclei at the fertilization check. Fertil Steril. 2010;94(3):965–70. doi: 10.1016/j.fertnstert.2009.04.018 19476942

[16] Capalbo A, Treff N, Cimadomo D, Tao X, Ferrero S, Vaiarelli A, et al. Abnormally fertilized oocytes can result in healthy live births: improved genetic technologies for preimplantation genetic testing can be used to rescue viable embryos in in vitro fertilization cycles. Fertil Steril. 2017;108(6):1007-1015.e3. doi: 10.1016/j.fertnstert.2017.08.004 28923286

[17] Li M, Dang Y, Wang Y, Li J, Liu P. Value of transferring embryos derived from monopronucleated (1PN) zygotes at the time of fertilization assessment. Zygote. 2020;28(3):241–6. doi: 10.1017/S096719942000009X 32192549

[18] Kemper JM, Liu Y, Afnan M, Mol BWJ, Morbeck DE. What happens to abnormally fertilized embryos? A scoping review. Reprod Biomed Online. 2023;46(5):802–7. doi: 10.1016/j.rbmo.2023.02.005 36997399

[19] Li M, Zhao W, Xue X, Zhang S, Shi W, Shi J. Three pro-nuclei (3PN) incidence factors and clinical outcomes: a retrospective study from the fresh embryo transfer of in vitro fertilization with donor sperm (IVF-D). Int J Clin Exp Med. 2015;8(8):13997–4003. 26550358 PMC4613043

[20] Chen Z, Yan J, Feng HL. Aneuploid analysis of tripronuclear zygotes derived from in vitro fertilization and intracytoplasmic sperm injection in humans. Fertil Steril. 2005;83(6):1845–8. doi: 10.1016/j.fertnstert.2004.11.076 15950663

[21] Canon C, Thurman A, Li A, Hernandez-Nieto C, Lee JA, Roth RM, et al. Assessing the clinical viability of micro 3 pronuclei zygotes. J Assist Reprod Genet. 2023;40(7):1765–72. doi: 10.1007/s10815-023-02830-y 37227570 PMC10352191

[22] Yalçınkaya E, Özay A, Ergin EG, Öztel Z, Özörnek H. Live birth after transfer of a tripronuclear embryo: An intracytoplasmic sperm injection as a combination of microarray and time-lapse technology. Turk J Obstet Gynecol. 2016;13(2):95–8. doi: 10.4274/tjod.45144 28913100 PMC5558346

[23] Takahashi H, Hirata R, Otsuki J, Habara T, Hayashi N. Are tri-pronuclear embryos that show two normal-sized pronuclei and additional smaller pronuclei useful for embryo transfer?. Reprod Med Biol. 2022;21(1):e12462. doi: 10.1002/rmb2.12462 35619657 PMC9126567

